# Editorial: The Genetic and Environmental Basis for Diseases in Understudied Populations

**DOI:** 10.3389/fgene.2020.559956

**Published:** 2020-09-23

**Authors:** Nicola Mulder, Zané Lombard, Mayowa Ojo Owolabi, Solomon Fiifi Ofori-Acquah

**Affiliations:** ^1^Computational Biology Division, Department Integrative Biomedical Sciences, IDM, University of Cape Town, Cape Town, South Africa; ^2^Division of Human Genetics, National Health Laboratory Service & School of Pathology, Faculty of Health Sciences, University of the Witwatersrand, Johannesburg, South Africa; ^3^Center for Genomic and Precision Medicine, University of Ibadan, Ibadan, Nigeria; ^4^West African Genetic Medicine Center, College of Health Sciences, University of Ghana, Accra, Ghana

**Keywords:** understudied populations, genetics, disease, GWAS, pharmacogenomics

Large-scale genomics research is costly, requiring significant resources for community engagement, participant recruitment, experimentation, and genomics data generation. Though the costs of genotyping and sequencing are decreasing, the large sample sizes required for genome wide association studies restrict such studies to researchers with significant funding and adequate resources. Until recently, these studies have been performed predominantly in European and other first world country populations, creating a bias in representation of global populations in public databases. Through a change in funding priorities for some major biomedical research funders, and a recognition of the need for diversity in genetic data, the balance has begun to shift, and under-represented populations are increasingly being included in genomics studies. Data from these under-represented populations have the potential to significantly alter our understanding of the genetic basis for human diseases in all populations, as they enable us to complete a picture which previously had major gaps. For example, inclusion of African populations, our oldest and most diverse populations, is providing important insights into human evolution and the origin of disease-related mutations.

For this Research Topic, we sought high quality research papers describing novel insights into genetic and environmental factors that impact disease risk, expression, prognosis, and treatment in understudied populations in human genomics research. Topics could include Population genetics, genome wide association studies, epigenetics, pharmacogenomics, environmental risk factors for diseases or gene-environment interactions in diseases. The final topic issue has 19 published articles covering various diseases studied in African and other previously under-represented populations.

Though not reporting specific studies, Shaffer et al. describe capacity development efforts in Mali to increase the number of trained bioinformaticians and data scientists able to analyse and interpret large-scale genomics data on local populations. Some of the papers describe novel methods or evaluation of existing methods for working on complex populations. For example, Schurz, Müller et al. evaluated the accuracy of three different imputation methods for multi-way admixed populations, using the South African Colored population as an example. Their findings demonstrate the importance of using an appropriate imputation software and reference panel containing populations that accurately represent ancestral populations for admixed individuals.

Fatumo et al. report the first GWAS in a Ugandan population for multivariate blood cell count phenotypes. The authors used both univariate and multivariate approaches and demonstrated that the multivariate approach has larger power and identifies additional loci. They report that performing a joint analysis of correlated phenotype simultaneously can provide new insights into complex traits that may not be identified in separate univariate analyses. However, there was an observation that highly correlated traits may also inflate *p*-values. New candidate loci for several blood cell count parameters were found illustrating the need for conducting GWAS in non-European populations to better understand the genetics of blood cell physiology.

There are a number of articles reporting on studies in African populations, most commonly on cohorts in South Africa. Vorster et al. describe a multiplex ligation-dependent probe amplification analysis to determine the cause of spinal muscular atrophy (SMA) in Black South African patients. Though no significant pathogenic CNVs were detected, they found discordant copy numbers of exons which suggest complex rearrangements that may affect the *SMN1* gene. Their study reiterates the fact that the genetic determinants of SMA in some individuals from this population group differ from those identified previously in other populations. Infectious diseases such as HIV and TB are prevalent in African populations, and mental illness is on the increase. Kalungi et al. sought to identify associations between relative telomere length and internalizing mental disorders, such as depressive disorders, anxiety disorders, and post-traumatic stress disorders, among young HIV-infected Ugandan individuals. A longer relative telomere length was found in individuals with these orders than in age- and sex-matched controls, and they concluded that though the length was not the cause of the disorders, the disorders were causing accelerated cellular aging.

In another Africa GWAS study, motivated by the epidemiological evidence that males are more affected by tuberculosis (TB) than females, Schurz, Kinnear et al. reported the first TB host susceptibility genome-wide association study (GWAS) with a specific focus on sex-stratified autosomal analysis and the X chromosome. Although the results are only nominally indicative of association, it does highlight the significance of the X chromosome in TB susceptibility, and the importance of considering ascertainment bias in genotyping arrays when selecting appropriate genotyping tools for undertaking studies in understudied populations.

Looking at environmental impact, Joubert et al. systematically reviewed the important progress and promising opportunities in environmental health research in Africa. Literature describing harmful health effects of metals, pesticides, and dietary mold represented a context unique to Africa. However, cardiovascular and respiratory health endpoints impacted by air pollution were comparable to observations in other countries. Air pollution exposures unique to Africa were dust and specific occupational exposures. Investigations of environmental exposures with distinct routes of exposure, unique co-exposures and co-morbidities, combined with the extensive genomic diversity in Africa in the context of gene-environment studies may lead to the identification of novel mechanisms underlying complex disease and promising potential for translation to global public health.

In line with this prospect, Boua et al. examined gene-smoking interactions with carotid intima-media thickness (cIMT) to identify potential drivers for atherosclerosis risk in West-African populations of the AWI-Gen Study. They identified new gene-smoking interaction variants for cIMT within the previously described RCBTB1 region and the novel regulatory region of TBC1D8. *In silico* functional analysis suggested the involvement of genes implicated in biological processes related to cell or biological adhesion and regulatory processes in gene-smoking interactions with cIMT.

Precision medicine and pharmacogenomics was a strong theme in several of the featured publications. In a paper from outside the African continent, Nagar et al. surveyed pharmacogenomic variants in two populations in Colombia, Antioquia and Chocó, with differing ancestries. They found that some pharmacogenomic variants have unusually high minor allele frequencies and differentiation according to ancestral contributions. These included variants with toxicity and dosing implications. As a result, the authors developed a cost-effective allele-specific PCR assay to test for relevant variants to inform healthcare decisions. In another paper, O'Connell et al. investigated the potential role of regulatory genes in antipsychotic treatment response in South African schizophrenia patients. Seven candidate genes showed significant expression level changes and four variants within these genes were significantly associated with treatment response. Compared to previously reported studies, two of these variants are identified as lying within eQTLs that impact brain gene expression, providing promising evidence that these may potentially serve as biomarkers of antipsychotic treatment response in the future.

## Author Contributions

All authors listed have made a substantial, direct and intellectual contribution to the work, and approved it for publication.

## Conflict of Interest

The authors declare that the research was conducted in the absence of any commercial or financial relationships that could be construed as a potential conflict of interest.

